# First In Silico Screening of Insect Molecules for Identification of Novel Anti-Parasitic Compounds

**DOI:** 10.3390/ph15020119

**Published:** 2022-01-19

**Authors:** Tom L. Gallinger, Samuel Y. Aboagye, Wiebke Obermann, Michael Weiss, Arnold Grünweller, Carlo Unverzagt, David L. Williams, Martin Schlitzer, Simone Haeberlein

**Affiliations:** 1 Department of Pharmaceutical Chemistry, Philipps University Marburg, 35032 Marburg, Germany; tom.gallinger@pharmazie.uni-marburg.de (T.L.G.); wiebkeobermann@staff.uni-marburg.de (W.O.); arnold.gruenweller@staff.uni-marburg.de (A.G.); schlitzer@staff.uni-marburg.de (M.S.); 2 Department of Microbial Pathogens and Immunity, Rush University Medical Center, Chicago, IL 60612, USA; Samuel_Y_Aboagye@rush.edu (S.Y.A.); David_Williams@rush.edu (D.L.W.); 3 Bioorganic Chemistry, University of Bayreuth, 95440 Bayreuth, Germany; Michael1.Weiss@uni-bayreuth.de (M.W.); Carlo.Unverzagt@uni-bayreuth.de (C.U.); 4 Institute of Parasitology, Justus Liebig University Giessen, 35392 Giessen, Germany

**Keywords:** *Schistosoma mansoni*, thioredoxin glutathione reductase, insect compounds, in silico screening, molecular docking

## Abstract

Schistosomiasis is a neglected tropical disease caused by blood flukes of the genus *Schistosoma*. In silico screenings of compounds for the identification of novel anti-parasitic drug candidates have received considerable attention in recent years, including the screening of natural compounds. For the first time, we investigated molecules from insects, a rather neglected source in drug discovery, in an in silico screening approach to find novel antischistosomal compounds. Based on the Dictionary of Natural Products (DNP), we created a library of 1327 insect compounds suitable for molecular docking. A structure-based virtual screening against the crystal structure of a known druggable target in *Schistosoma mansoni*, the thioredoxin glutathione reductase (SmTGR), was performed. The top ten compounds predominantly originated from beetles and were predicted to interact particularly with amino acids in the doorstop pocket of SmTGR. For one compound from a jewel beetle, buprestin H, we tested and confirmed antischistosomal activity against adult and juvenile parasites in vitro. At concentrations with anti-parasitic activity, we could also exclude any unspecific cytotoxic activity against human HepG2 cells. This study highlights the potential of insect molecules for the identification of novel antischistosomal compounds. Our library of insect-derived molecules could serve not only as basis for future in silico screenings against additional target proteins of schistosomes, but also of other parasites.

## 1. Introduction

Up to 50% of all approved drugs are classified as natural products or were at least inspired by one [1]. This is not surprising, given the high structural diversity of natural products that makes them an outstanding source of novel molecular scaffolds in drug discovery. A growing body of literature highlights the potential of plant-derived, microbial, fungal and marine products also in the treatment of neglected tropical diseases (NTDs) [2]. Even though insects represent, with an estimated 5.5 million species, the most diverse group of animals on earth [3], their potential as a source of therapeutic compounds has barely been explored. In fact, science has just scratched the surface of the insects’ biochemical diversity. Evolution created an overwhelming diversity of compounds produced by insects for offensive, defensive and social purposes, a diversity that has been honed by 480 million years of trial and error [4,5]. The finding of antitumor and antiviral activities of insect products indeed highlights their potential in modern medicine [6]. We like to pursue the hypothesis that insect-derived compounds can serve as a source for new drugs against medically important parasites, for many of which new therapeutic options are heavily needed because either no or only one approved drug exists, or drug resistance has emerged [7].

One of these parasitic diseases is schistosomiasis, which is caused by blood flukes (class Trematoda) of the genus *Schistosoma.* This NTD is transmitted in 78 countries, and recent estimates of the World Health Organization suggest that more than 236 million people require treatment [8]. Patients suffer from severe morbidity that may affect their ability to work. Symptoms include anemia, lassitude, and growth stunting in children, and in some cases infection can result in death. Consequently, the economic and health effects are tremendous [9]. Schistosomes have a complex life cycle that involves a vertebrate as a final host and an invertebrate intermediate host (freshwater snails). Humans are infected when they come in contact with the infectious larval stages in contaminated freshwater, which penetrate the skin and then mature and reside in blood vessels of their host. Because of the lack of an effective vaccine, the control of schistosomiasis relies on mass drug administration programs with praziquantel, the currently only available drug [10]. Likely because of its use since the 1970s, decreasing sensitivity to praziquantel in field studies has been repeatedly described and resistance in the laboratory has been demonstrated [11,12,13]. It is, therefore, clear that new antischistosomal drugs are urgently needed.

Targeting the antioxidant pathway of schistosomes has emerged as one promising strategy. Maintaining the redox balance is crucial to the blood parasite because of its aerobic environment and to protect it from redox active molecules of the host’s immune response [14]. In platyhelminths including *S*. *mansoni*, thiol redox homeostasis depends on a single enzyme, thioredoxin-glutathione reductase (TGR, Smp_048430). SmTGR binds NADPH at its binding site, which transfers electrons to the FAD cofactor and then the proximal Cys residues located in the redox active center. The electrons are then transferred to the carboxy-terminal selenocysteine–cysteine redox pair. Reducing equivalents are finally transferred to both thioredoxin and glutathione (GSH), two major contributors to the maintenance of redox balance in eukaryotes [15,16,17]. Small-molecule inhibitors and RNA interference against SmTGR have proven that this enzyme is essential for parasite survival in vitro and in vivo [16]. Thus, TGR represents one of the most promising targets for antischistosomal chemotherapy to date. Indeed, high throughput screenings (HTS) against recombinant schistosomal TGR successfully identified new chemical structures with activity against the parasites [18,19].

Next to HTS, virtual screening plays an important role in lead discovery processes because it is rapid, cost-effective, and considerably reduces the number of compounds to be screened in whole-organism assays. In the structure-based screening approach, databases comprising chemical structures of thousands to millions of compounds are screened against the active site residues of a target protein to predict molecules with bioactivity [20]. Moreover, in the schistosomiasis research field, the use of in silico screening has been pushed forward in recent years, including screenings of chemical libraries or fragments against SmTGR [18,19,21,22,23]. Despite the reinforced interest in natural products for antischistosomal lead discovery [24,25], natural products and in particular insect products have been basically neglected in such screenings. Motivated by our pilot studies that revealed antischistosomal activity of ladybird- and assassin-bug-derived alkaloid and venom compounds [26,27], we wanted to close this gap and performed a virtual screening of a library of more than one thousand insect-derived compounds. As a protein target with a known vital function for schistosomes, we have chosen SmTGR. Because of the lack of a public or commercial database that provides structure codes of insect molecules with information on their stereochemistry, which is mandatory for molecular docking, we first established a pipeline to create such a library based on the Dictionary of Natural Products [28]. Several rounds of docking were performed to identify potential SmTGR inhibitors, and visual inspection along with analyses using the PLIP (Protein–Ligand Interaction Profiler) web tool [29] predicted several key residues involved in the interactions. Our study highlights the usefulness of insects for the identification of unexplored, biologically active chemical scaffolds with antischistosomal potential. To our knowledge, this is the first time that a large-scale in silico screening of insect molecules has been performed.

## 2. Results

### 2.1. Creation of a Library of Insect Compounds Suitable for Docking Studies

**Figure 1:**
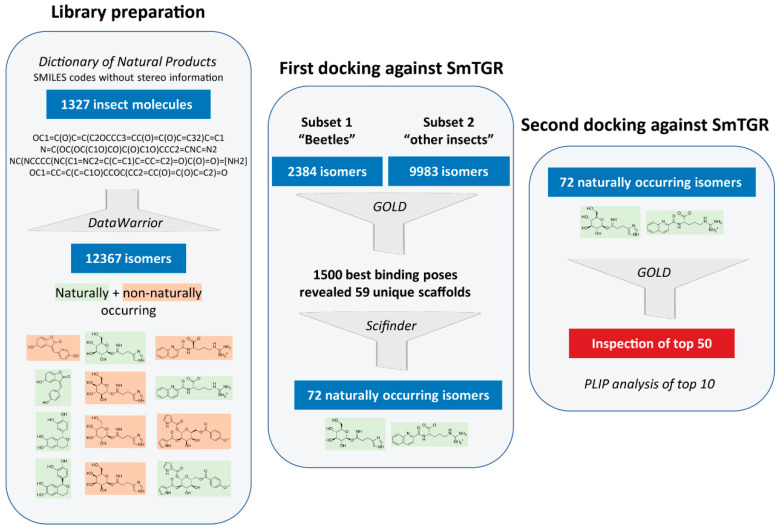
Virtual screening workflow used for identifying insect molecules with potential inhibitory activity against thioredoxin glutathione reductase (TGR) of *Schistosoma mansoni*.

At the start of this project, no library containing insect substances was available that could be reasonably applied for docking studies. Hence, the first step was to create such a library. As a basis for this, the Dictionary of Natural Products (DNP) [28] was used, from which SMILES codes of substances originating from the taxonomic orders Coleoptera (beetles), Diptera (true flies), Lepidoptera (moths and butterflies), Hymenoptera (wasps, bees, ants and sawflies), Hemiptera (true bugs) and other insects were obtained (Figure 1). The major downside was that the SMILES codes provided did not contain any stereo information. Since the manual adjustment of the stereocenters of all 1327 molecules would have been too time-consuming and in order to not exclude any naturally occurring isomers in the following docking analyses, all possible stereo isomers were generated in an automated process from the initially obtained SMILES codes. This increased the original number of 1327 molecules to 12,367 molecules. At the penultimate step of our virtual screening pipeline, isomers that do not occur naturally were retrospectively removed from the hit list of molecules in order to obtain a final hit list of naturally occurring molecules.

### 2.2. Prioritization of SmTGR Ligand-Binding Pockets

**Figure 2:**
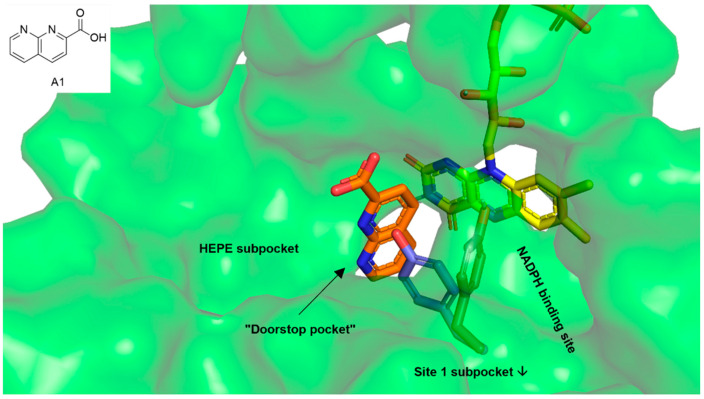
The green surface shows the cavity within thioredoxin glutathione reductase (TGR) of *Schistosoma mansoni* (6FP4) addressed in the docking analysis. The cavity includes the doorstop pocket as well as the adjacent HEPE (1-(2-hydroxyethyl)piperazine) subpocket and the NADPH binding site that partially overlaps with the Site 1-subpocket [30]. Yellow sticks—cofactor FAD, orange sticks—1,8-naphthyridine-2-carboxylate (**A1**) occupying the doorstop pocket, gray—Tyr296 in the 1,8-naphthyridine-2-carboxylate (**A1**) bound state, blue—Tyr296 in the NADPH bound state (superimposed from SmTGR in complex with NADPH, 2X99).

We decided to address the so-called “doorstop pocket” of SmTGR in our docking analyses. This pocket was identified by a combination of structural and functional studies and is adjacent to the NADPH binding site. Binding of 1,8-naphthyridine-2-carboxylate (**A1**, orange sticks in Figure 2) into this pocket prevents Tyr296 from rotating (blue sticks—NADPH bound SmTGR, magenta sticks—**A1** bound SmTGR), which is needed for NADPH entry and enzyme activity. This way, the occupied pocket acts as a doorstop for the entry of NADPH [30]. Since the insect molecules from our library comprised both fragment-like and large complex natural products, and additional HEPE (1-(2-hydroxyethyl)piperazine) and Site 1 subpockets were identified adjacent to the doorstop pocket [23,30], docking analyses were performed throughout the cavity that is built by all pockets (green surface in Figure 2).

### 2.3. Docking Results

#### 2.3.1. Docking Predicts SmTGR-Inhibiting Insect Compounds

**Figure 3:**
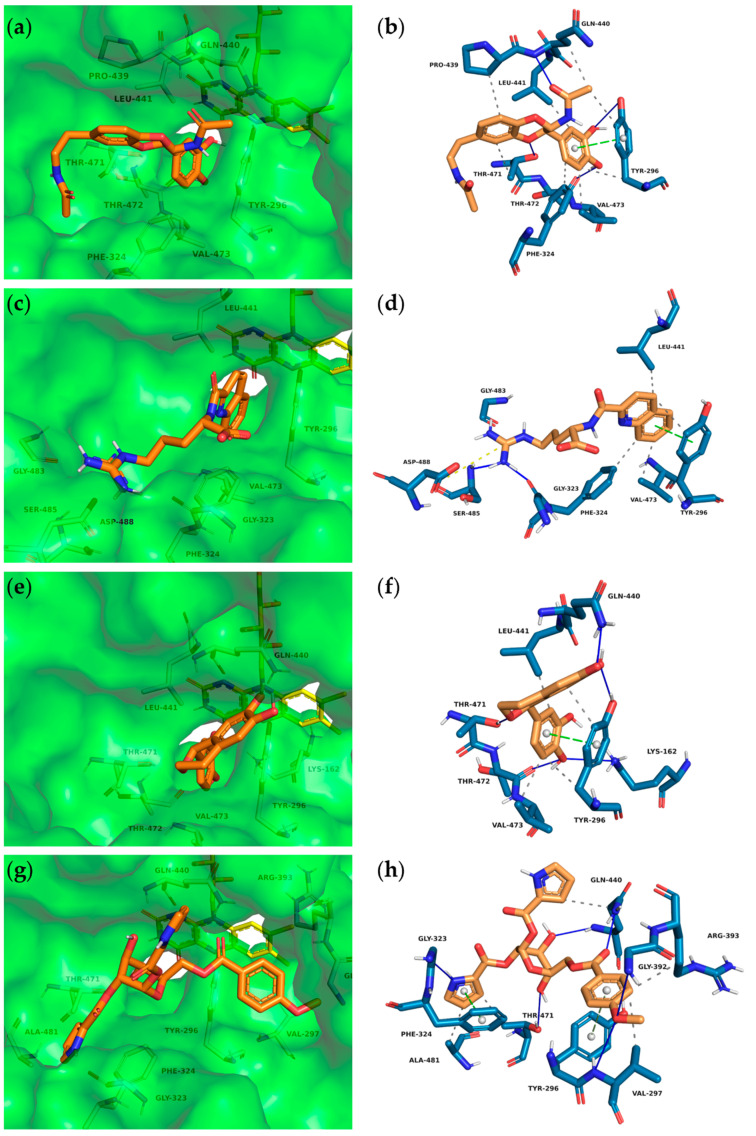
Interactions between insect molecules and SmTGR. Docking poses (left) and PLIP analyses (right) of molecules **1** (**a**) + (**b**), **4** (**c**) + (**d**), **5** (**e**) + (**f**) and **7** (**g**) + (**h**). Orange sticks—insect molecules, yellow sticks—FAD, dashed gray lines—hydrophobic interactions, blue lines—hydrogen bonds, dashed green lines—π-stacking (parallel), dashed gray lines—π-stacking (edge to face), orange dashed lines—salt bridge.

Two consecutive docking analyses were carried out. The first was intended to identify the highest-ranking scaffolds from all 12,367 isomers generated. Therefore, the substances from Coleoptera (“beetles subset”) and Diptera, Lepidoptera, Hymenoptera, Hemiptera and other insects (“other subset”) were docked and the best binding poses of the two subsets were analyzed. The smaller “beetles subset” (2384 molecules) was examined separately to test this approach before screening the other larger subset. A detailed evaluation of the 500 best binding poses of the “beetles subset” and 1000 of the “other subset” revealed that often different stereoisomers of the same molecules were present. However, only 59 unique scaffolds, disregarding the stereo information, were among these 1500 binding poses. This initial docking analysis was followed by a literature search using *SciFinder*^n^ [31] in order to identify the naturally occurring stereoisomers of these 59 scaffolds. For some of the scaffolds, more than one isomer was identified, so that we ended with a final list of 72 naturally occurring isomers. Subsequently, the second round of docking was performed with these 72 isomers. The 50 top-ranking molecules were visually inspected and the ten most promising compounds occupying the doorstop pocket and/or having a convincing binding pose were selected and additionally analyzed with the PLIP-tool [29,32]. These most promising compounds are shown in Table 1 and selected docking poses are presented in Figure 3.

The compounds obtained in the docking comprise different structures from different groups of insects. Compounds **1, 2** and **3** are *N*-acetyldopamine dimers and were first isolated from the cicada *Cryptotympana tustulata* F_ABR_. Isomer **1** was also found in stink bugs and stink beetles as well as dung beetles [33,34,35,36,37], whereas **2** and **3** might not naturally occur, but were characterized after potential racemization during the extraction [35]. Amide **4** is a defensive alkaloid that was isolated from the ladybird *Subcoccinella vigintiquatuorpunctata* [38]. The isochromans **5** and **6** were isolated from the stink beetle *Blaps japanensis* and are called blapsin B [39]. Buprestin H (**7**) is an acyl glucose derivative isolated from the pine borer *Chalcophora mariana*, a European jewel beetle [40]. The phenylacetic acid ester **8** is known as blapsin A and was derived from *B. japanensis* [39]. The imidate **9** is known as polybioside and is a neuroactive venom isolated from the social wasp *Polybia paulista* [41]. Marginalin (**10**) was isolated from pygidial bladders of the water beetle *Dysticus marginalis* and was later assigned to be the *E* isomer [42]. Taken together, the majority of top-ranking insect molecules from the docking analysis originated from beetles, whereas individual molecules originated from hemipteran or hymenopteran insects.

#### 2.3.2. Predicted Interactions within the SmTGR Cavity

The binding poses of the selected top ten compounds and additional PLIP analyses revealed the most likely interactions with the various subpockets of SmTGR. Overall, the hydroxy-substituted aromatic residues of the majority of compounds showed comparable binding modes and were occupying the doorstop pocket (**1**, **2**, **3**, **5**, **6**, **8**, **10)**. These include hydrogen bonds between the phenolic hydroxy groups and the amino acids Lys162, Thr472, Thr471, Tyr296 and Glu300. Note that not every interaction was predicted for every ligand. Furthermore, at least one aromatic ring of **1–6** and **8–10** was predicted to have hydrophobic interactions and π–π interactions with Tyr296 and/or Phe324 while occupying the doorstop pocket. Additionally, hydrogen bonds with the cofactor FAD are conceivable for **1**, **5** and **6** (not shown in the PLIP analyses). In the following, we will describe the most important interactions for four compounds (Figure 3), which display a representative variety of binding modalities. The full dataset obtained after PLIP analyses and the docking poses of all compounds listed in Table 1 can be found in the supplementary information.

The central amide moiety of ligands **1** (Figure 3a,b), **2** and **3** showed hydrogen bonding with the main and/or the side chain of Gln440. Furthermore, one ether oxygen of **1** is in close proximity (3.0 Å) to the OH-moiety of Thr471 and thus is capable of accepting a hydrogen bond. Due to the stereocenter, enantiomers **5** (Figure 3e,f) and **6** address different amino acids through hydrogen bonds. Ligand **5** binds with its phenolic OH-groups to the side chain Gln440-NH_2_ and Tyr296-OH and thus keeps the Tyr296 in this position. Similar to 1,8-naphthyridine-2-carboxylate (**A1**) in SmTGR (6FP4, Figure 2), the quinoline ring of **4** is occupying the doorstop pocket through π–π interactions (parallel displaced, charge transfer) and hydrophobic effects. In contrast to **A1**, ligand **4** is not able to serve as a hydrogen bond acceptor for the main chain Gln440-NH because of the distance of 4.2 Å to the carbonyl group. Instead, the arginine residue interacts in a salt bridge with the carboxylate of As488 and in a hydrogen bond with the carbonyl oxygen of Gly323. This chain is thereby also targeting the HEPE subpocket. The carboxylate of ligand **4** is not addressed, but only extends into the aqueous phase.

Among the 50 highest-ranking ligands, compounds of the buprestin family appeared frequently. As a representative, buprestin H (**7**) (Figure 3g,h) is discussed. In contrast to the other ligands, buprestin H is not directly occupying the doorstop pocket, but covers it by extending from the HEPE to the NADPH binding pocket. One pyrrole-2-carboxylate unit is able to form π–π interactions (parallel displaced) with Phe324 and the methoxy benzene ring with Tyr296. The pyrrole-NH also forms a hydrogen bond with the carbonyl oxygen of Gly323. The main chain Gln440-NH is addressed through one glycosidic hydroxy group and the side chain Gln440-NH_2_ through the benzoic acid carbonyl group. In summary, most of the insect compounds interacted with residues in the doorstop pocket with their (di)hydroxy substituted aromatic rings. Additionally, several compounds addressed the NADPH or the HEPE subpockets.

### 2.4. Validation of Buprestin H Activity against Schistosoma mansoni In Vitro

#### 2.4.1. Activity against Adult Worms

**Figure 4:**
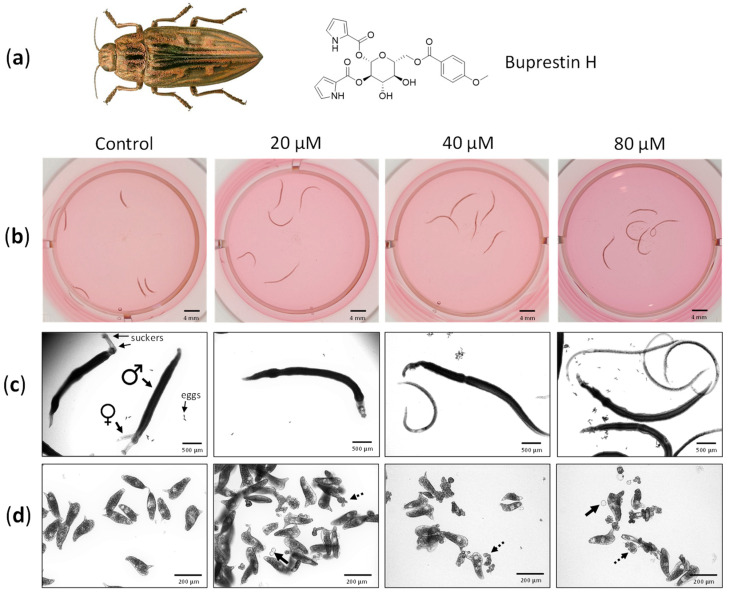
Buprestin H has antischistosomal activity against adult parasites. (**a**) Adult worm couples of *Schistosoma mansoni* were treated with buprestin H originally found in the pine borer *Chalcophora mariana*, the European jewel beetle, at different concentrations (20, 40, 80 µM) or with the solvent ethanol as a control (amount equivalent to 80 µM of buprestin H). (**b**) Bodies of the worms were extended by buprestin H. Examples after 48 h treatment are shown. (**c**) Buprestin H at concentrations of 40 and 80 µM caused a reduction in vitality. Control worms are in pairs and attached with their suckers, while buprestin H caused a separation of male and female worms and detachment. (**d**) At 20 µM and above, buprestin H led to a production of fewer eggs by female worms and an increasing abundance of abnormally shaped eggs, free-floating oocytes (solid arrows) and vitelline cells (dashed arrows). (**b**–**d**) Representative images after six days culture and from two independent experiments with 5 worm couples per condition are shown. Beetle image taken from Biodiversity Map with permission [43].

We aimed to validate the docking results by testing compounds against *S. mansoni*. We selected buprestin H, which was originally discovered in the jewel beetle *C. mariana*, the European pine borer (Figure 4a) [40], because several acyl glucoses were among the top 50 of the predicted SmTGR-binding compounds, and buprestin H was the only compound available for tests. Although no inhibition of NAPDH-binding could be confirmed in an established SmTGR assay [16] in concentrations up to 60 µM (data not shown), we could demonstrate activity of buprestin H against the parasite. Adult *S. mansoni* were treated with 20–80 µM of buprestin H for a period of six days and their vitality was assessed daily. With concentrations as low as 20 µM, the body length was found considerably extended compared to control worms already 24 h after the start of the treatment and stayed extended throughout the culture period (Figure 4b). Treatment with 40 and 80 µM of buprestin H caused in addition a reduction in vitality compared to healthy control worms, which started after 24 h and became more severe until the end of the experiment.

While healthy female and male worms form a couple, with their bodies united, and attach with their suckers to the culture dish, buprestin H affected both vitality parameters (Figure 4c). Moreover, the motility of treated worms was considerably lower compared to controls (Supplementary Videos S1–S3). Finally, buprestin H decreased parasite reproduction at 20 µM with even more pronounced effects at higher concentrations: egg numbers released by the female worms during culture declined, and an increasing fraction of eggs were abnormally shaped. Free-floating oocytes and vitellocytes were also observed, which are usually packed into eggs inside the female’s body (Figure 4d).

#### 2.4.2. Activity against Juvenile Worms and Cytotoxic Potential

**Figure 5:**
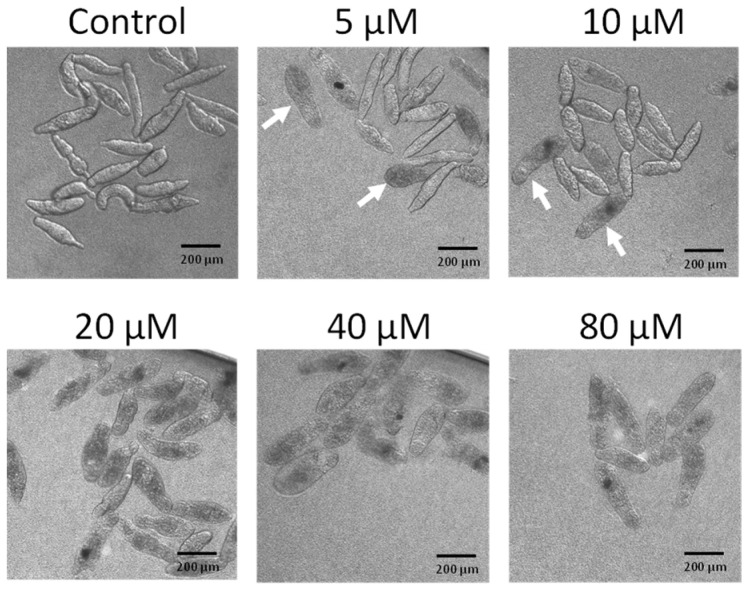
Buprestin H kills juvenile worms (schistosomula). Schistosomula of *Schistosoma mansoni* were treated with buprestin H at concentrations from 5 to 80 µM or with the solvent ethanol (amount equivalent to 80 µM buprestin H) for a period of 72 h. Dark, bloated individuals were dead and were occasionally found with 5 and 10 µM (arrows) and exclusively at 20 µM and above. Representative images of about 100 schistosomula per condition and from two independent experiments are shown.

To determine whether buprestin H has activity only against adult worms or also younger parasite stages, we tested buprestin H against schistosomula. Buprestin H showed an even higher activity at lower concentrations against this juvenile stage compared to the adult stage. First dead, bloated schistosomula were found with 5 µM within three days of treatment, while all parasites were dead with 20 µM (Figure 5).

**Figure 6:**
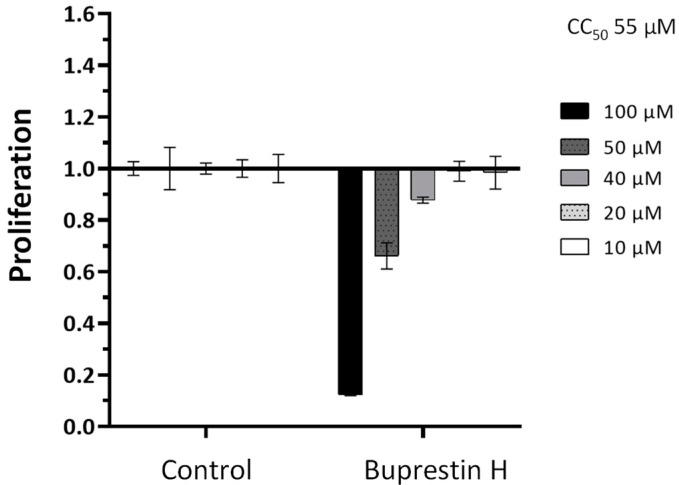
Quantification of cytotoxicity by buprestin H against eukaryotic cells. HepG2 cells were treated with buprestin H at different concentrations (10 to 100 µM) or equivalent amounts of the solvent ethanol for a period of 48 h. Cell proliferation was quantified in a WST-1 assay and normalized to the control conditions. Means with standard error of the mean from four experiments are shown. The CC_50_ (cytotoxic concentration) calculated from the data was 55 µM.

To determine whether the antischistosomal effect is rather specific or results from a general cytotoxicity, we additionally performed in vitro viability tests against the human HepG2 cell line. Five different concentrations from 10 to 100 µM were tested in a WST-1 assay that measures cell proliferation. This yielded a CC_50_ (cytotoxic concentration) of 55 µM (Figure 6), a concentration higher than the lowest active antischistosomal concentrations. Taken together, by employing in silico docking studies of insect-derived molecules, we identified a new compound with antischistosomal activity.

## 3. Discussion

Our study aimed at evaluating whether structure-based virtual screening of insect molecules might be a promising strategy for the identification of new compounds with antischistosomal activity. To this end, we first created a pipeline that allows the use of the DNP insect database as a basis for a virtual screening library. We showed that among 1327 insect molecules, several were predicted to bind into the doorstop pocket and adjacent pockets of schistosomal TGR, and thereby confirmed the suitability of this insect-based approach. That the majority of the top-ranking insect molecules originated from beetles is not surprising, given the fact that beetles represent by far the largest group of insects [3] and consequently the largest fraction in the DNP database. Most compounds have roles as a defense system for the insect, such as a component (**9**) of neuroactive venom from a social wasp, an antimicrobial and antifungal compound (**10**) from the great diving beetle, and a defensive alkaloid (**4**) from a ladybird that acts as ant deterrent [38,41,42].

### 3.1. Advances Achieved in Virtual Screening of Insect Molecules

Virtual screenings of compound libraries to identify compounds with activity against schistosomes have received attention in recent years [44,45,46,47]. Moreover, against SmTGR, such screenings have been successful in identifying active molecules [22,48]. These screens typically involved 150,000 and more molecules. With respect to natural compounds, however, only very few studies were conducted, such as one screen against TGR of the liver fluke *Fasciola gigantica*, another trematode parasite [49]. To our knowledge, our study is the first one to apply insect molecules in a large-scale structure-based virtual screening in the schistosome research field and beyond.

Previous studies have docked individual insect molecules, such as silkworm peptides or a bee toxin, against putative target structures to find treatment options against diabetes or cardiovascular diseases [50,51]. The lack of large-scale screenings is not surprising given the fact that a library of insect molecules suitable for docking studies is not available. The existing natural compound libraries typically used in virtual screenings to date, such as the Specs natural product library and libraries from the ZINC database or the Database of Traditional Chinese Medicine, mainly comprise plant-derived, microbial, fungal and marine products but no insect molecules, or do not provide a search option for insect-derived molecules [52,53,54]. The latter is implemented in the Dictionary of Natural Products [28], but has the problem of not being optimized for virtual screenings (e.g., SMILES codes without stereo information, elaborate download procedure). We solved this problem by manual curation of the obtained codes and introduction of the stereo information through *DataWarrior* [55] to gain an insect library suitable for virtual screenings. The pipeline developed in our study will be helpful to enable future insect-based molecular docking studies as well as virtual screenings of compound libraries from other organism sources that currently lack necessary stereo information. Priority in such screenings should be given a priori to small-molecule-like compounds that are more likely to succeed in preclinical studies. In our study, we excluded peptides larger than tripeptides and other structurally or chemically unfavorable molecules. Furthermore, in silico analysis of pharmaceutically relevant properties, such as carcinogenicity and biodegradability, by ADMET prediction could help in speeding up the process of finding favorable insect molecules with drug-likeness [56].

### 3.2. Predicted Molecular Interactions between Insect Molecules and SmTGR

We also determined key residues possibly involved in the interaction of the selected insect molecules with a known druggable pocket within SmTGR. Hydrogen bonding was described as the main interaction for stabilizing the ligand–TGR complex. One of the best studied SmTGR inhibitors binding at the doorstop pocket is 1,8-naphthyridine-2-carboxylate (**A1**). This naphthyridine forms H-bonds with Gln440 and Thr471 within this pocket, which indirectly prevents Tyr296 from rotating [30]. Our docking analyses predicted the same hydrogen bonding for several of the top ten insect molecules (e.g., ligands **1, 2, 3** and **6**) and these might, therefore, interfere in a similar way with NADPH entry. Additionally, several insect molecules directly addressed the key residue Tyr296 through H-bonding and π–π interactions, even if the latter are not ideal since the aromatic rings involved are electron-rich in both cases [57]. Ligand **4** shows clear structural similarity to **A1** and, according to our expectations, exhibits a comparable binding mode in our docking studies. In contrast to the other ligands, buprestin H (**7**) was not directly occupying the interior of the doorstop pocket but covered it by extending from the HEPE subpocket to the NADPH binding pocket. We can only speculate that interactions that do not directly involve the doorstop pocket might be too weak and, therefore, have prevented buprestin H from successfully inhibiting NAPDH oxidation in an SmTGR enzyme assay. When we tested buprestin H at concentrations at which effects were seen in the phenotypic screens against *S. mansoni*, even at 60 µM, no inhibitory effect against the enzyme was found. Considering an IC_50_ of the SmTGR-inhibiting compound **A1** of 1.1 mM [30], it might be necessary to test buprestin H at similar high concentrations to achieve inhibitory effects. However, high concentrations of ethanol, used as solvent for buprestin H in the assay, do not allow such tests. Thus, the formal proof that buprestin H is a TGR inhibitor has still to be made. Beyond this, in silico tools that predict the most probable protein targets of small molecules could reveal whether buprestin H might inhibit other enzyme targets beyond TGR, although one needs to bear in mind that such predictions use protein databases of humans or model organisms, and not schistosomes [58].

### 3.3. Buprestin H as Antischistosomal Compound

The biological function of buprestin H in its source organism, a European jewel beetle, is unknown. Unlike the major buprestins A and B from the Australian counterpart, buprestin H did not display an insect-deterrent activity [40,59]. We found a general impairment of vitality when *S. mansoni* was treated with buprestin H, which in adult worms typically results in detachment of the suckers, release of the female from the male’s “embrace”, and a decrease of body movements. Exceptional was the elongation of body length, which is rarely found upon compound exposure of worms. One study has described a body elongation of adult worms by mefloquine [60], which acts as a protein synthesis inhibitor [61]. Furthermore, an RNAi screen of 2216 genes in *S. mansoni* found body elongation phenotypes for some genes involved in protein ubiquitination (ubiquitin ligase E2, Smp_103710) or deubiquitination (DUB, Smp_069960) [62]. At least in mammals, the cellular redox status is known to modulate protein ubiquitination by reversible S-thiolation, and certain ubiquitin ligases were proposed as possible Grx substrates [63]. Thus, modulation of (de)ubiquitination downstream of SmTGR may be one factor provoking the observed body elongation, although this remains speculation at this point.

### 3.4. Hurdles to Overcome for In Vitro Screens of Insect Molecules

The antischistosomal activity of buprestin H against adult worms was less than 1,8-naphthyridine-2-carboxylate [30], which might be related to the fact that buprestin H is not as efficiently slipping into the doorstop pocket. For this reason, it would be of great interest to test the other insect molecules against the parasite that occupied the doorstop pocket in our docking analysis. The problem with insect molecules is their availability: as natural products, they are often structurally very complex and sophisticated protocols for synthesis need to be established, and extraction from the natural source might be difficult to achieve. For these two reasons, we have not yet been able to complete more comprehensive in vitro tests. This is at the same time a call to the research community, to the pharmaceutical industry and commercial library providers to recognize the value and to create chemical libraries for insect compounds for in vitro test purposes, as has been done with other types of natural products. New pipelines are needed, ranging from the large-scale rearing of prioritized insect species to compound purification from sources such as insect hemolymph or venom. Recently, new biotechnology and biodiversity institutions, such as the Fraunhofer Institute for Bioresources, have started to tackle this demand. This will enable scientists to explore the full breadth of this gigantic natural resource and efficiently use insects as starting points not only for novel anti-parasitic drugs, but—considering the enormous biodiversity of insects and their bioactive substances—a wider range of diseases.

## 4. Materials and Methods

### 4.1. Data Set Preparation

SMILES codes of compounds originating from insects were obtained from the Dictionary of Natural Products (DNP, https://dnp.chemnetbase.com/faces/chemical/ChemicalSearch.xhtml, accessed between 25 November 2019 and 23 December 2019). The data of the available insect orders Coleoptera (beetles), Diptera, Lepidoptera, Hymenoptera, Hemiptera and “other insects” were individually accessed. Datasets of compounds with the following properties or missing SMILES codes were excluded before download: peptides larger than tripeptides, aliphatic compounds (if C_x_H_x_ only), fatty acids and relating esters or alcohols if acyclic. The obtained datasets (xls-files) were checked, duplicates removed, and incorrect SMILES codes, which were not correctly transformed into a 2D structure in the next step, were corrected. Subsequently, the SMILES codes (1327 in total) were transformed into 2D structures using *DataWarrior* (version v05.02.01, https://openmolecules.org/datawarrior/index.html, accessed on 02 April 2020) [55] and the protonation state of the acidic and basic groups was inspected and corrected if necessary. In the following step, the structures were energy minimized using the following settings: algorithm: random, low energy bias; initial torsions: from crystallographic database; minimize energy: MMFF94s+ forcefield. By doing this, all possible stereo isomers (in total 12,367) were generated. This step was necessary because the SMILES codes obtained from the DNP did not contain stereo information. *DataWarrior* files (.dwar) containing the compound datasets of the individual insect orders can be found in the supplementary information. Finally, the datasets of Diptera, Lepidoptera, Hymenoptera, Hemiptera and other insects were merged and duplicates removed. The resulting dataset (“other dataset”) and the beetle dataset were saved as sdf-file (version 2) and used for docking.

### 4.2. Docking Analyses

Docking analyses were done using the crystal structure 6FP4 of SmTGR, which is the only available structure showing the crucial switch of Tyr296 [30]. Flexible docking with the software GOLD (v2020.2.0) [64] was performed as follows: The structure was loaded and hydrogens were added. Water molecules in the cavity (see below) were extracted, all other water molecules were deleted and the “toggle mode” was activated (GOLD decides whether an interaction with a water molecule is considered or not). Then, ligands and cofactors were deleted, with FAD being an exception. The binding site was defined as the cavity in a 10 Å radius around 1,8-naphthyridine-2-carboxylate (activated “detect cavity mode”). ASP was chosen as fitness function and the top-ranked solutions for the best ligands were kept depending on the experiment (1000—“other dataset”, 500—beetles dataset, 50—docking round 2, top 50). A literature search using *SciFinder*^n^ [31] was applied to identify naturally occurring stereoisomers. To validate the docking procedure, 1,8-naphthyridine-2-carboxylate was docked prior to the docking of the insect substances. The docking pose was found to be comparable to that of the crystal structure (6FP4) (see Supplementary Figure S11).

### 4.3. Visual Inspection

Visual inspection was performed using PyMOL (The PyMOL Molecular Graphics System, version 2.4 Schrödinger, LLC, Munich, Germany) and assessment of the top-ranked solutions was based on the values given for typical distances and angles as published by Bissantz, Kuhl and Stahl [65]. When considering the poses, the most important parameter was whether the ligand binds in the doorstop pocket.

### 4.4. PLIP Analyses

PLIP analyses were performed with the PLIP web tool [29,32] in the default settings.

### 4.5. Buprestin H

Buprestin H, an acyl glucose originally isolated from the jewel beetle *C. mariana* (Buprestidae), was chemically synthesized [40]. In brief, a triol precursor was reacted with *p*-anisic acid in a Mitsunobu reaction to form the *p*-methoxybenzoylated buprestin (buprestin H). The structural integrity was confirmed by LC-MS prior to testing.

### 4.6. Maintenance of Schistosoma mansoni

A Liberian strain of *S. mansoni* was maintained in the freshwater snail *Biomphalaria glabrata* as intermediate host and Syrian hamsters (*Mesocricetus auratus*) as final host (infected at 8 weeks old; Janvier Labs, France) [66,67]. Hamsters were infected by the “paddling method” [68] with 1750 cercariae. Adult worms were collected at 46 days p.i. by hepatoportal perfusion and cultured in supplemented M199 medium (Sigma-Aldrich, Schnelldorf, Germany; supplemented with 10% Newborn Calf Serum (NCS), 1% HEPES [1 M] and 1% ABAM-solution (10,000 units penicillin, 10 mg streptomycin and 25 mg amphotericin B per mL)) at 37 °C, 5% CO_2_ and humidified atmosphere.

### 4.7. Biological Evaluation on Schistosoma mansoni

Schistosomula were prepared from freshly shed cercariae following established protocols [69]. In brief, cercariae were transformed to schistosomula by mechanically shearing tails from heads by passing back and forth between two 10 mL syringes attached via a 22-gauge double-headed needle in ice-cold RPMI 1640 medium (Gibco, Thermo Fisher Scientific, Bremen, Germany). Schistosomula were collected and washed several times in the same medium and used for testing buprestin H within 2 h. Schistosomula were incubated at about 100 per well in a flat-bottomed 96-well plate in supplemented M199 medium (without HEPES) with buprestin H (5–80 µM) at 37 °C and 5% CO_2_ in a humidified atmosphere. Phenotypes were monitored after 3 d, and attention was paid to changes in movement, shape, translucence, and surface integrity. Adult worms were treated with buprestin H (20–80 µM) with 10 worm couples per well in a 6-well plate in supplemented M199 medium for up to 6 days. Phenotypes including separation of male and female worms, detachment of suckers from the bottom, and weakening of body movements were monitored daily. In addition, eggs laid by the female worms during in vitro culture were evaluated with respect to numbers and morphology. All control worms received the solvent ethanol in an amount equivalent to 80 µM buprestin H. Phenotypes were monitored using a Leica DM IL inverted microscope and x2.5 to x10 objective lens for adults and schistosomula, respectively.

### 4.8. Cytotoxic Measurements

Human liver hepatoma (HepG2) cells were obtained from American Type Culture Collection (ATCC HB-8065, Manassas, VA, USA) and were cultivated in Iscove’s modified Dulbecco medium (IMDM, Lonza) supplemented with 10% fetal calf serum (FBS Superior, Sigma-Aldrich) at 37 °C and 5% CO_2_. A WST-1 (Roche, Mannheim, Germany) assay was conducted to quantify cell proliferation. To this end, 2 × 10^4^ cells were seeded in 200 µL IMDM supplemented with 10% FCS in 96-well cell culture plates (Greiner-CELLSTAR, Sigma-Aldrich) and incubated for 24 h (37 °C, 5% CO_2_). Afterwards, medium was replaced by fresh medium containing 10 to 100 µM of buprestin H in ethanol. Ethanol-treated cells served as control. After another 48 h of cultivation, medium was again removed and 110 µL of 10% WST-1 reagent in phosphate-buffered saline (PBS, Gibco, Thermo Fisher Scientific) was added. Absorbance was measured at 450 nm after 2 h by using a Tecan Safire II (*n* = 4 for every concentration).

### 4.9. Ethics Statement

Animal experiments using Syrian hamsters as model hosts for *S. mansoni* were performed in accordance with the European Convention for the Protection of Vertebrate Animals used for experimental and other scientific purposes (revised Appendix A of ETS No 123). Experiments have been approved by the Regional Council (Regierungspraesidium) Giessen (V54-19 c 20/15 h 02 GI 18/10 Nr. A 14/2017).

## 5. Conclusions

Our study successfully demonstrated that the structure-based virtual screening of insect molecules represents a useful strategy to identify new compounds with antischistosomal activity. By using SmTGR as a known druggable target, ten potential inhibitors derived from insects were identified by molecular docking. As proof of concept, we tested and confirmed in vitro the antischistosomal activity of one compound. Although the availability of natural compounds derived from insects is currently limited, the identified compounds or partial motifs of these could serve as the basis for a structure-based development of SmTGR inhibitors in the future.

## Figures and Tables

**Table 1:** Structures, trivial names and origin of potential inhibitors of SmTGR identified by docking of insect molecules. Where known, a biological function or activity is also indicated.

No., Name	Structure	Origin	Function/Activity ^+^
**1, 2, 3**	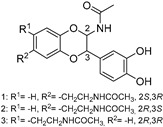	1: Cicada—*Cryptotympana tustulata* F_ABR_. (Hemiptera), Stink bug—*Cotidius chinensis*/*Aspongopus chinensis* (Hemiptera), Stink beetle—*Blaps japanensis*, Dung beetle—*Catharsius molossus* (Coleoptera) [33,34,35,36,37] 2/3: Cicada—*Cryptotympana tustulata* F_ABR_. (Hemiptera) [35] ^#^	Crude drug “zentai”, COX-2 inhibitor, whitening agent
**4**	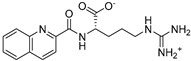	Ladybird—*Subcoccinella vigintiquatuorpunctata* (Coleoptera) [38]	Defensive alkaloid (ant deterrent)
**5 (*R*)**, **6 (*S*)** Blapsin B	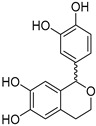	Stink beetle—*Blaps japanensis* (Coleoptera) [39]	Inhibitor of 14-3-3 protein-protein interactions, antioxidant
**7** Buprestin H	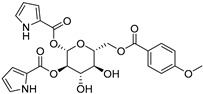	Jewel beetle—*Chalcophora mariana* (Coleoptera) [40]	Unknown
**8** Blapsin A	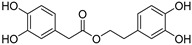	Stink beetle—*Blaps japanensis* (Coleoptera) [39]	Inhibitor of 14-3-3 protein-protein interactions
**9** Polybioside	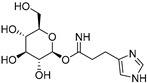	Social wasp—*Polybia paulista* (Hymenoptera) [41]	Component in neuroactive venom
**10** Marginalin	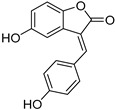	Water beetle, *Dytiscus marginalis* (Coleoptera) [42]	Pygidial bladders coloring substance

^+^ References can be found in the organism column. ^#^ The authors indicated that the initial 2*S*, 3*R* compound might have racemized during extraction.

## Data Availability

The data presented in this study are available in the supplementary information.

## Supplementary Materials

The following supporting information can be downloaded at: https://www.mdpi.com/article/10.3390/ph15020119/s1, Figures S1–S10 and Tables S1–S10: Docking poses and PLIP analyses of insect molecules **1**–**10** against SmTGR. Figure S11: Comparison of the docking pose of **A1** with the crystal structure (6FP4). *DataWarrior* files (.dwar) containing the compound datasets of Coleoptera, Diptera, Lepidoptera, Hymenoptera, Hemiptera and “other insects”. Video S1: Normal motility of control-treated adult *Schistosoma mansoni*. Video S2: Reduced motility of adult *Schistosoma mansoni* treated with 40 µM of buprestin H for 6 d. Video S3: Reduced motility of adult *Schistosoma mansoni* treated with 80 µM of buprestin H for 6 d.
