# Supplementary material for: First In Silico Screening of Insect Molecules for Identification of Novel Anti-Parasitic Compounds
Source: Pharmaceuticals (Basel). 2022 Jan 19;15(2):119. doi: 10.3390/ph15020119 (PMC8877563; doi:10.3390/ph15020119)
Supplement: Supplementary file 1 [file pharmaceuticals-15-00119-s001.zip › pharmaceuticals-1551494_supplementary tables and figures.pdf]

Supplementary Information

First *In Silico* Screening of Insect Molecules for Identification of Novel Anti-Parasitic Compounds

Tom L. Gallinger [1], Samuel Y. Aboagye [2], Wiebke Obermann [1], Michael Weiss [3], Arnold Grünweller [1], Carlo Unverzagt [3], David L. Williams [2], Martin Schlitzer [1], Simone Haeberlein [4,*]

[1] Department of Pharmaceutical Chemistry, Philipps University Marburg, Marburg, Germany
[2] Department of Microbial Pathogens and Immunity, Rush University Medical Center, Chicago, Illinois, United States
[3] Bioorganic Chemistry, University of Bayreuth, Bayreuth, Germany
[4] Institute of Parasitology, Justus Liebig University Giessen, Giessen, Germany
* Correspondence: simone.haeberlein@vetmed.uni-giessen.de; Tel.: +49-6419938476

Table of Contents **Page**

Docking Results of Ligand **1** 2

Docking Results of Ligand **2** 3

Docking Results of Ligand **3** 4

Docking Results of Ligand **4** 5

Docking Results of Ligand **5** 6

Docking Results of Ligand **6** 7

Docking Results of Ligand **7** 8

Docking Results of Ligand **8** 9

Docking Results of Ligand **9** 10

Docking Results of Ligand **10** 11

Redocking of 1,8-naphthyridine-2-carboxylate (**A1**) 12

Docking Results of Ligand 1

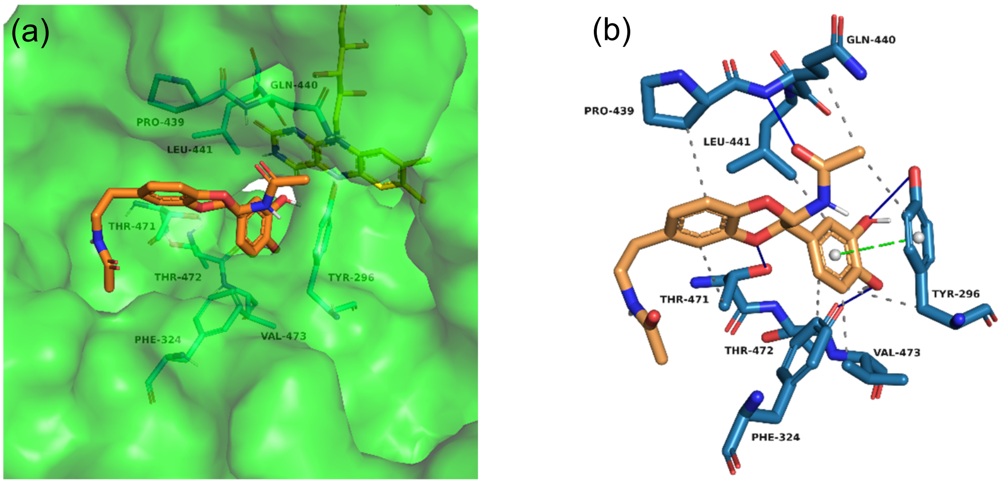

Figure S1. Interactions between **1** and SmTGR displayed as docking pose (a) and PLIP analysis (b). The part of the molecule occupying the doorstop pocket is predicted to interact with THR471 and 472 and TYR296 by hydrogen bonding and π-π interactions (TYR296) as well as hydrophobic interactions (TYR296, PHE324). The central amide moiety shows hydrogen bonding with the main side chain of GLN440. Orange sticks – insect molecule, yellow sticks – FAD, dashed grey lines – hydrophobic interactions, blue lines – hydrogen bonds, dashed green lines – π-stacking (parallel), orange dashed lines – salt bridge.

Table S1: Interactions between **1** and SmTGR as observed in the PLIP analysis.

Hydrophobic Interactions

Index	Residue	AA	Distance	Ligand Atom	Protein Atom
1	296A	TYR	3.68	18	4503
2	296A	TYR	3.64	4	4508
3	324A	PHE	3.20	14	4933
4	439A	PRO	3.94	12	6717
5	440A	GLN	3.56	4	6731
6	441A	LEU	2.76	13	6751
7	471A	THR	3.91	15	7209
8	473A	VAL	3.25	18	7238

Hydrogen Bonds

Index	Residue	AA	Distance H-A	Distance D-A	Donor Angle	Protein donor?	Side chain	Donor Atom	Acceptor Atom
1	296A	TYR	3.16	3.78	122.61	×	✓	20 [O2]	4510 [O3]
2	440A	GLN	2.10	2.93	162.03	✓	×	6727 [Nam]	1 [O3]
3	471A	THR	2.30	3.04	147.30	✓	✓	7210 [O3]	9 [O2]
4	472A	THR	1.90	2.85	164.42	×	×	23 [O2]	7221 [O2]

π-Stacking

Index	Residue	AA	Distance	Angle	Offset	Stacking Type	Ligand Atoms
1	296A	TYR	3.69	20.46	0.33	P	10, 13, 14, 17, 18, 21

Docking Results of Ligand 2

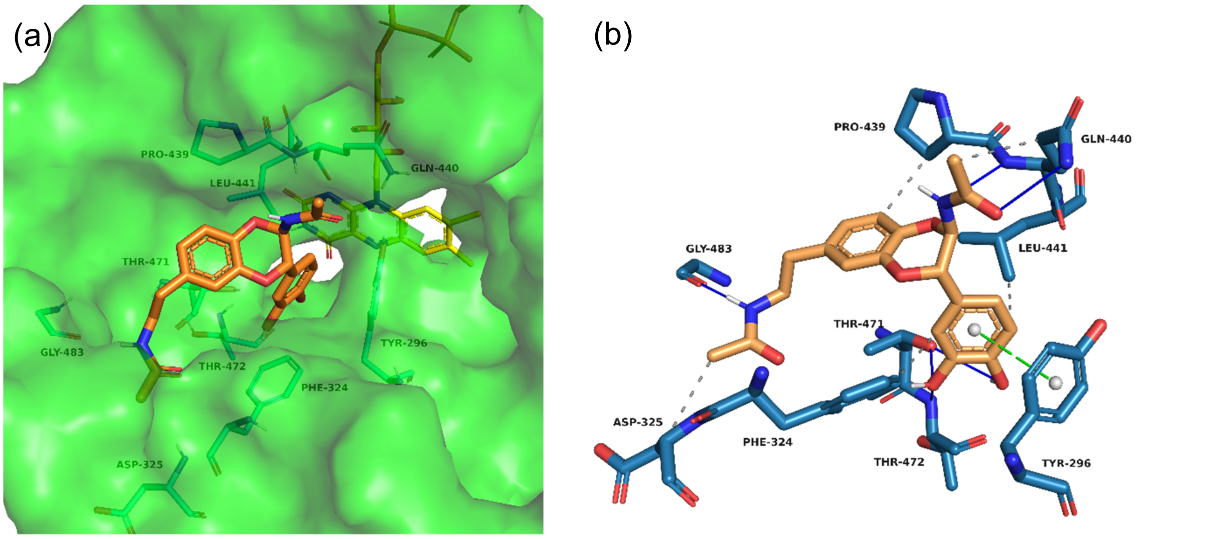

Figure S2. Interactions between **2** and SmTGR displayed as docking pose (a) and PLIP analysis (b). Compared to the interactions described for **1** in SI Figure 1, analysis of ligand **2** revealed that its terminal amide-NH is able to form a hydrogen bond with the main chain GLY483 carbonyl oxygen but not with TYR296. This ligand is thereby also targeting the HEPE subpocket. Orange sticks – insect molecule, yellow sticks – FAD, dashed grey lines – hydrophobic interactions, blue lines – hydrogen bonds, dashed green lines – π-stacking (parallel), orange dashed lines – salt bridge.

Table S2: Interactions between **2** and SmTGR as observed in the PLIP analysis.

Hydrophobic Interactions

Index	Residue	AA	Distance	Ligand Atom	Protein Atom
1	324A	PHE	3.18	13	4933
2	325A	ASP	4.00	28	4947
3	439A	PRO	3.16	12	6717
4	440A	GLN	3.67	4	6732
5	441A	LEU	3.14	18	6751

Hydrogen Bonds

Index	Residue	AA	Distance H-A	Distance D-A	Donor Angle	Protein donor?	Side chain	Donor Atom	Acceptor Atom
1	440A	GLN	2.66	3.48	161.04	✓	×	6727 [Nam]	3 [N3]
2	440A	GLN	2.42	3.16	144.88	✓	✓	6734 [Nam]	1 [O3]
3	471A	THR	2.17	2.57	109.30	✓	✓	7210 [O3]	20 [O2]
4	471A	THR	2.83	3.47	123.60	×	✓	23 [O2]	7210 [O3]
5	472A	THR	3.82	4.10	102.68	✓	×	7218 [Nam]	20 [O2]
6	483A	GLY	1.98	2.83	139.15	×	×	25 [N3]	7382 [O2]

π-Stacking

Index	Residue	AA	Distance	Angle	Offset	Stacking Type	Ligand Atoms
1	296A	TYR	4.02	28.77	1.39	P	10, 13, 14, 17, 18, 21

Docking Results of Ligand 3

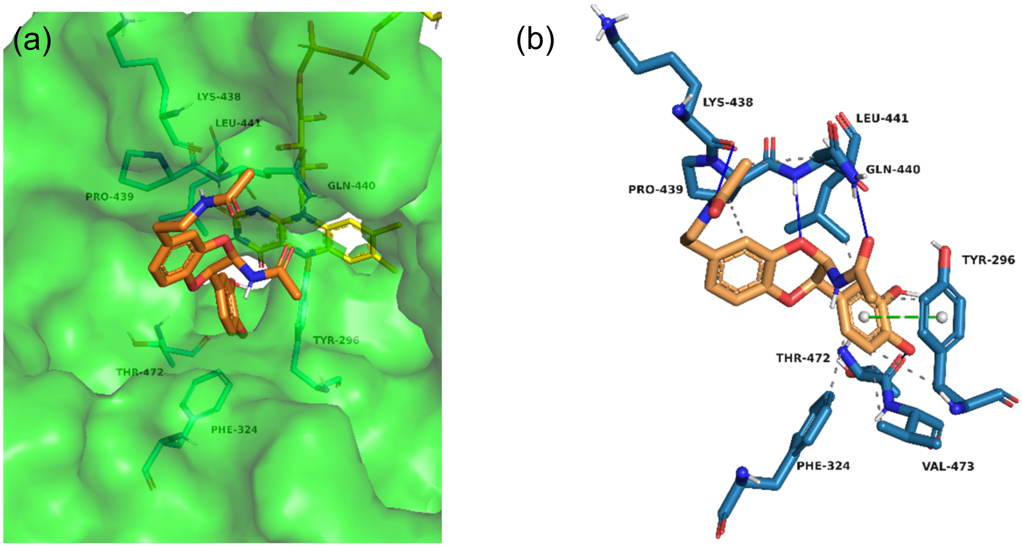

Figure S3. Interactions between **3** and SmTGR displayed as docking pose (a) and PLIP analysis (b). Compared to the docking pose described for **1** in SI Figure 1, analysis of ligand **3** revealed that this isomer does not stick out to the left (HEPE subpocket) or right (NADPH subpocket), but rather upwards. By doing this, it is able to undergo additional hydrogen bonds with GLN440-NH and LYS438. Orange sticks – insect molecule, yellow sticks – FAD, dashed grey lines – hydrophobic interactions, blue lines – hydrogen bonds, dashed green lines – π-stacking (parallel), orange dashed lines – salt bridge.

Table S3: Interactions between **3** and SmTGR as observed in the PLIP analysis.

Hydrophobic Interactions

Index	Residue	AA	Distance	Ligand Atom	Protein Atom
1	296A	TYR	3.69	18	4503
2	296A	TYR	3.51	4	4508
3	324A	PHE	3.09	14	4933
4	439A	PRO	3.78	12	6717
5	440A	GLN	3.92	28	6732
6	441A	LEU	2.46	13	6751
7	473A	VAL	3.75	18	7238

Hydrogen Bonds

Index	Residue	AA	Distance H-A	Distance D-A	Donor Angle	Protein donor?	Side chain	Donor Atom	Acceptor Atom
1	438A	LYS	2.28	3.24	156.93	×	×	25 [N3]	6694 [O2]
2	440A	GLN	2.12	2.96	166.07	✓	×	6727 [Nam]	6 [O2]
3	440A	GLN	2.33	3.18	169.81	✓	✓	6734 [Nam]	1 [O3]
4	472A	THR	2.07	3.01	163.50	×	×	24 [O2]	7221 [O2]

π-Stacking

Index	Residue	AA	Distance	Angle	Offset	Stacking Type	Ligand Atoms
1	296A	TYR	3.82	18.26	0.67	P	10, 13, 14, 17, 18, 22

Docking Results of Ligand 4

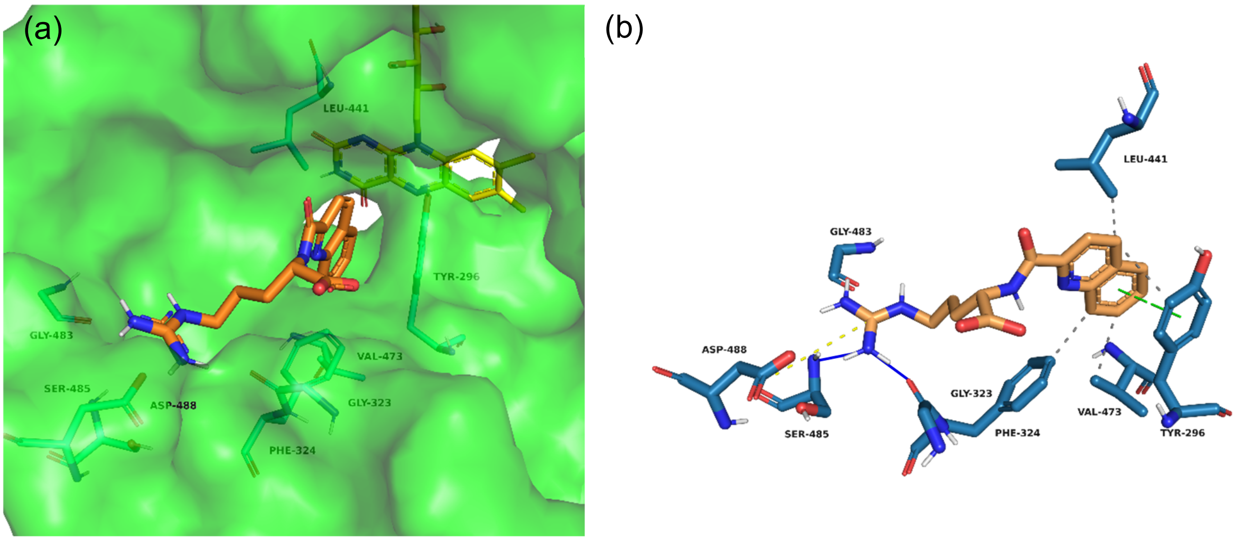

Figure S4. Interactions between **4** and SmTGR displayed as docking pose (a) and PLIP analysis (b). The quinoline ring of **4** is occupying the doorstop pocket through π-π interactions (parallel displaced, charge transfer) and hydrophobic effects. The arginine residue interacts in a salt bridge with the carboxylate of ASP488 and in a hydrogen bond with the carbonyl oxygen of GLY323. This chain is thereby also targeting the HEPE subpocket. Orange sticks – insect molecule, yellow sticks – FAD, dashed grey lines – hydrophobic interactions, blue lines – hydrogen bonds, dashed green lines – π-stacking (parallel), orange dashed lines – salt bridge.

Table S4: Interactions between **4** and SmTGR as observed in the PLIP analysis.

Hydrophobic Interactions

Index	Residue	AA	Distance	Ligand Atom	Protein Atom
1	296A	TYR	3.53	11	4496
2	324A	PHE	3.25	16	4921
3	441A	LEU	2.97	11	6739
4	473A	VAL	3.39	19	7226

Hydrogen Bonds

Index	Residue	AA	Distance H-A	Distance D-A	Donor Angle	Protein donor?	Side chain	Donor Atom	Acceptor Atom
1	323A	GLY	1.81	2.69	142.21	×	×	24 [Ng+]	4907 [O2]
2	323A	GLY	3.22	3.95	132.23	×	×	20 [Ng+]	4907 [O2]
3	483A	GLY	2.70	3.23	112.80	×	×	23 [Ng+]	7370 [O2]
4	485A	SER	3.14	3.92	152.43	✓	×	7393 [Nam]	24 [Ng+]

π-Stacking

Index	Residue	AA	Distance	Angle	Offset	Stacking Type	Ligand Atoms
1	296A	TYR	3.60	20.08	0.37	P	10, 15, 16, 18, 19, 21

Salt Bridges

Index	Residue	AA	Distance	Protein positive?	Ligand Group	Ligand Atoms
1	488A	ASP	5.05	×	Guanidine	20, 24, 23

Docking Results of Ligand 5

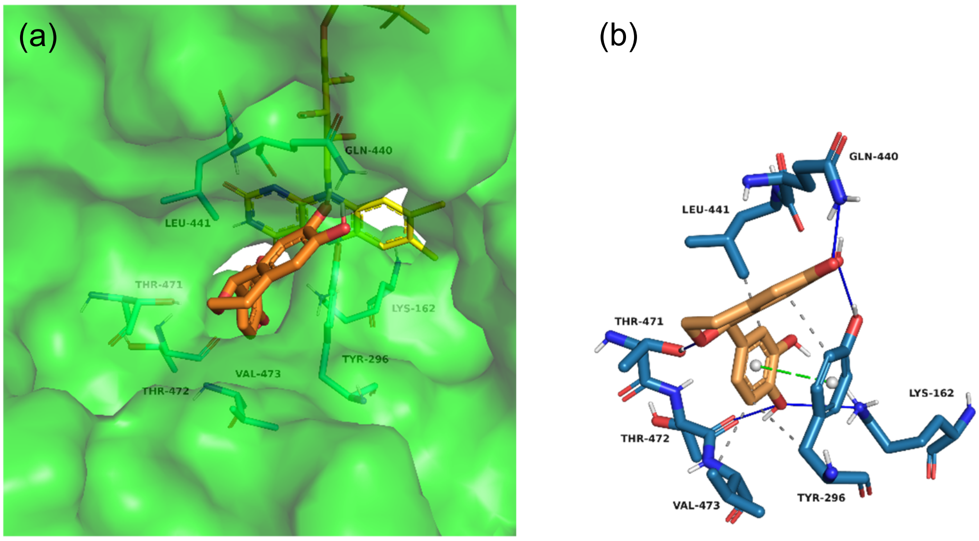

Figure S5. Interactions between **5** and SmTGR displayed as docking pose (a) and PLIP analysis (b). The part of the molecule occupying the doorstop pocket is predicted to interact with THR471 and 472 and TYR296 by hydrogen bonding and π-π interactions (TYR296) as well as hydrophobic interactions (TYR296). Ligand **5** binds with its phenolic OH-groups to the side chain GLN440-NH_2 and TYR296-OH and thus keeps the TYR296 in this position. Orange sticks – insect molecule, yellow sticks – FAD, dashed grey lines – hydrophobic interactions, blue lines – hydrogen bonds, dashed green lines – π-stacking (parallel), orange dashed lines – salt bridge.

Table S5: Interactions between **5** and SmTGR as observed in the PLIP analysis.

Hydrophobic Interactions

Index	Residue	AA	Distance	Ligand Atom	Protein Atom
1	296A	TYR	3.72	14	4485
2	296A	TYR	3.09	8	4490
3	441A	LEU	2.73	9	6733
4	473A	VAL	3.38	14	7220

Hydrogen Bonds

Index	Residue	AA	Distance H-A	Distance D-A	Donor Angle	Protein donor?	Side chain	Donor Atom	Acceptor Atom
1	162A	LYS	3.27	3.78	119.35	✔	✔	2397 [N3+]	20 [O2]
2	296A	TYR	2.17	2.98	161.98	✔	✔	4492 [O3]	16 [O2]
3	440A	GLN	1.72	2.46	143.27	✔	✔	6716 [Nam]	19 [O2]
4	471A	THR	3.20	4.04	176.87	✔	✔	7192 [O3]	1 [O3]
5	472A	THR	1.67	2.49	140.04	×	×	20 [O2]	7203 [O2]

π-Stacking

Index	Residue	AA	Distance	Angle	Offset	Stacking Type	Ligand Atoms
1	296A	TYR	3.58	9.72	0.27	P	5, 9, 10, 13, 14, 18

Docking Results of Ligand 6

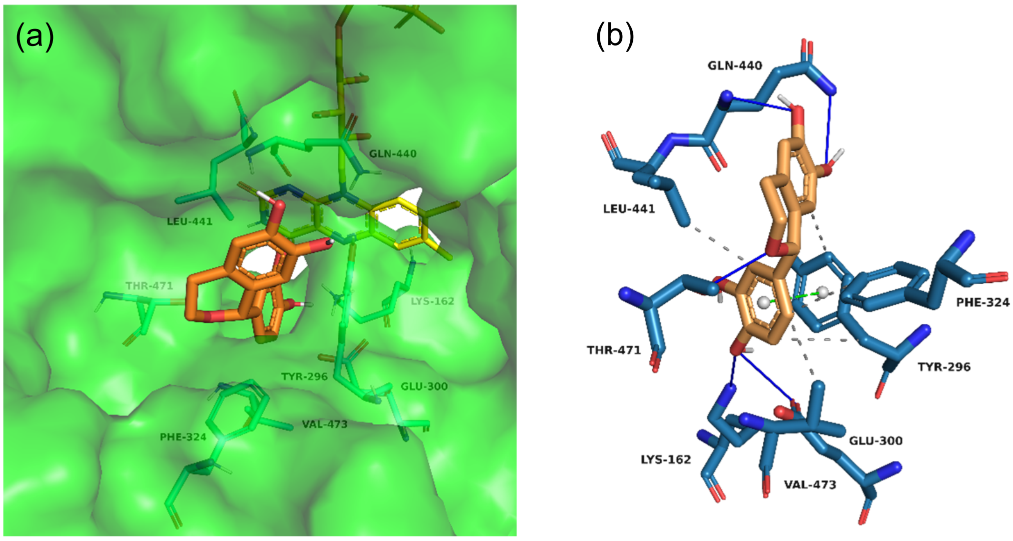

Figure S6. Interactions between **6** and SmTGR displayed as docking pose (a) and PLIP analysis (b). Occupation of the doorstop pocket is comparable to **5** but ligand **6** sticks out towards the HEPE subpocket and interacts with the main chain of GLN440. Orange sticks – insect molecule, yellow sticks – FAD, dashed grey lines – hydrophobic interactions, blue lines – hydrogen bonds, dashed green lines – π-stacking (parallel), orange dashed lines – salt bridge.

Table S6: Interactions between **6** and SmTGR as observed in the PLIP analysis.

Hydrophobic Interactions

Index	Residue	AA	Distance	Ligand Atom	Protein Atom
1	296A	TYR	3.64	14	4485
2	296A	TYR	3.21	8	4490
3	324A	PHE	3.64	10	4915
4	441A	LEU	3.19	9	6733
5	473A	VAL	3.27	10	7220

Hydrogen Bonds

Index	Residue	AA	Distance H-A	Distance D-A	Donor Angle	Protein donor?	Side chain	Donor Atom	Acceptor Atom
1	162A	LYS	2.75	3.19	112.17	✔	✔	2397 [N3+]	20 [O2]
2	300A	GLU	3.29	4.09	140.69	×	✔	20 [O2]	4554 [O3]
3	440A	GLN	2.82	3.68	174.98	✔	✔	6716 [Nam]	16 [O2]
4	440A	GLN	2.01	2.80	152.20	✔	×	6709 [Nam]	19 [O2]
5	471A	THR	1.97	2.79	164.95	✔	✔	7192 [O3]	1 [O3]

π-Stacking

Index	Residue	AA	Distance	Angle	Offset	Stacking Type	Ligand Atoms
1	296A	TYR	3.44	14.79	0.52	P	5, 9, 10, 13, 14, 18

Docking Results of Ligand 7

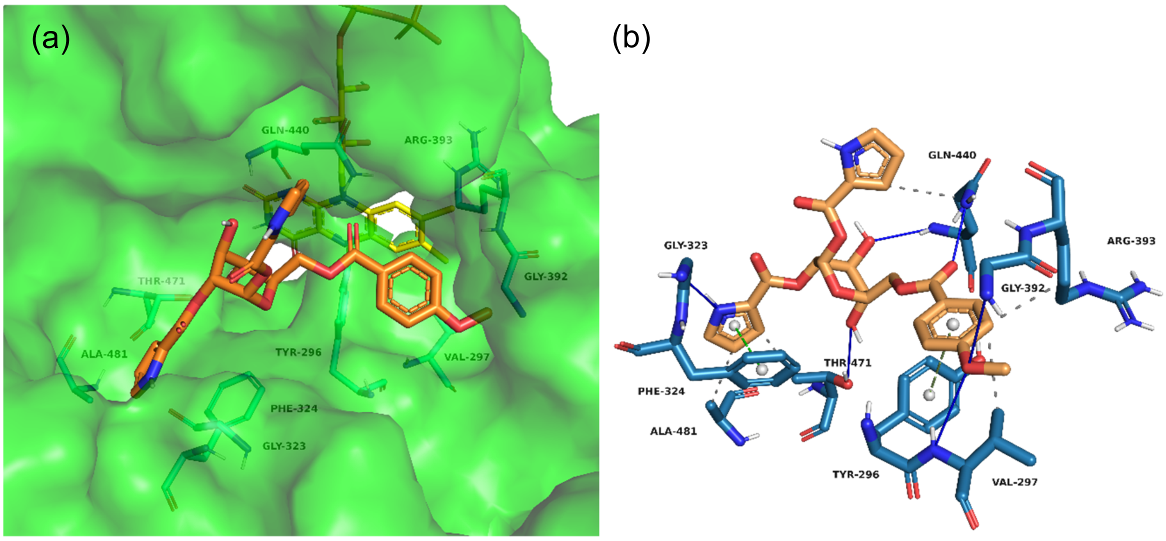

Figure S7. Interactions between **7** and SmTGR displayed as docking pose (a) and PLIP analysis (b). One pyrrole-2-carboxylate unit of ligand **7** is able to form π-π interactions (parallel displaced) with PHE324 and the methoxy benzene ring with TYR296. The pyrrole-NH also forms a hydrogen bond with the carbonyl oxygen of GLY323. The main chain GLN440-NH is addressed through one glycosidic hydroxy group and the side chain GLN440-NH_2 through the benzoic acid carbonyl group. Thus, **7** is not directly occupying the doorstop pocket, but covers it by extending from the HEPE to the NADPH binding pocket. Orange sticks – insect molecule, yellow sticks – FAD, dashed grey lines – hydrophobic interactions, blue lines – hydrogen bonds, dashed green lines – π-stacking (parallel), orange dashed lines – salt bridge.

Table S7: Interactions between **7** and SmTGR as observed in the PLIP analysis.

Hydrophobic Interactions

Index	Residue	AA	Distance	Ligand Atom	Protein Atom
1	297A	VAL	3.62	9	4538
2	393A	ARG	3.43	9	6035
3	440A	GLN	3.71	28	6744
4	471A	THR	3.60	34	7221
5	481A	ALA	3.92	36	7374

Hydrogen Bonds

Index	Residue	AA	Distance H-A	Distance D-A	Donor Angle	Protein donor?	Side chain	Donor Atom	Acceptor Atom
1	297A	VAL	3.32	4.05	144.28	✓	×	4532 [Nam]	17 [O2]
2	323A	GLY	2.01	2.98	159.83	×	×	33 [Npl]	4931 [O2]
3	392A	GLY	3.45	3.94	119.28	✓	×	6023 [Nam]	17 [O2]
4	440A	GLN	2.45	3.23	151.85	✓	×	6739 [Nam]	20 [O3]
5	440A	GLN	1.98	2.72	144.43	✓	✓	6746 [Nam]	1 [O3]
6	471A	THR	2.98	3.78	162.33	✓	✓	7222 [O3]	15 [O3]

π-Stacking

Index	Residue	AA	Distance	Angle	Offset	Stacking Type	Ligand Atoms
1	296A	TYR	4.42	67.21	1.89	T	4, 6, 7, 9, 10, 13
2	324A	PHE	3.93	26.50	0.52	P	30, 33, 34, 35, 36

Docking Results of Ligand 8

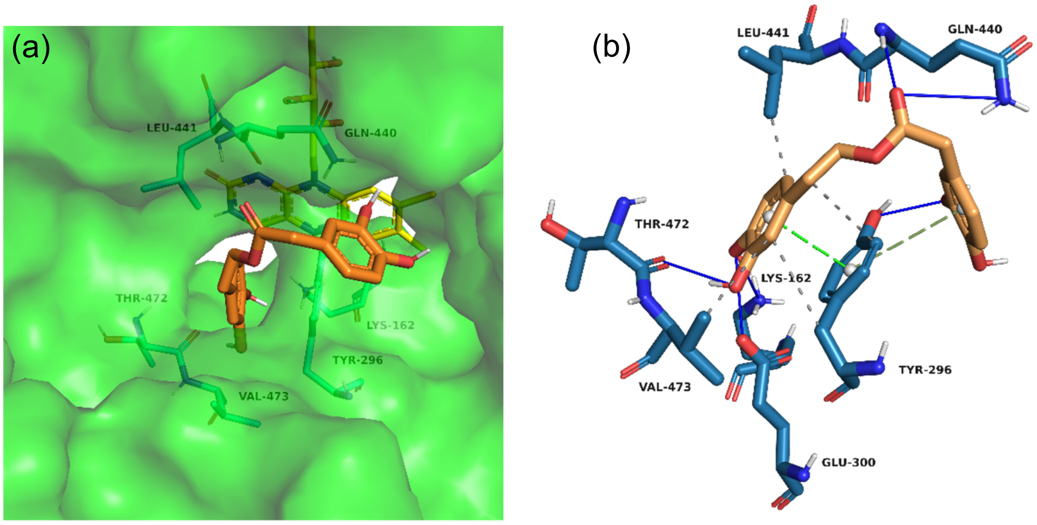

Figure S8. Interactions between **8** and SmTGR displayed as docking pose (a) and PLIP analysis (b). The part of the molecule occupying the doorstop pocket is predicted to interact with THR472 and TYR296 by hydrogen bonding and π-π interactions (TYR296) as well as hydrophobic interactions (TYR296). Analysis of ligand **8** revealed an additional π-π interaction with TYR296 (edge to face) and is thereby extending into the NADPH binding site. Additionally, the carbonyl oxygen of ligand **8** showed a further hydrogen bonding with the main chain GLN440-NH as well as the meta hydroxy group with TYR296-OH, while the para hydroxy group remained unclaimed. Orange sticks – insect molecule, yellow sticks – FAD, dashed grey lines – hydrophobic interactions, blue lines – hydrogen bonds, dashed green lines – π-stacking (parallel), dashed gray lines – π-stacking (edge to face), orange dashed lines – salt bridge.

Table S8: Interactions between **8** and SmTGR as observed in the PLIP analysis.

Hydrophobic Interactions

Index	Residue	AA	Distance	Ligand Atom	Protein Atom
1	296A	TYR	3.75	13	ID of protein carbon atom
2	296A	TYR	3.97	7	4494
3	441A	LEU	2.74	14	6737
4	473A	VAL	3.88	13	7224

Hydrogen Bonds

Index	Residue	AA	Distance H-A	Distance D-A	Donor Angle	Protein donor?	Side chain	Donor Atom	Acceptor Atom
1	162A	LYS	3.00	3.51	118.63	✓	✓	2401 [N3+]	22 [O2]
2	296A	TYR	2.12	2.74	129.95	✓	✓	4496 [O3]	15 [O2]
3	300A	GLU	2.47	3.30	142.92	×	✓	22 [O2]	4559 [O-]
4	440A	GLN	2.01	2.85	164.27	✓	×	6713 [Nam]	1 [O3]
5	440A	GLN	3.33	4.00	136.73	✓	✓	6720 [Nam]	1 [O3]
6	472A	THR	2.04	2.99	164.74	×	×	20 [O2]	7207 [O2]

π-Stacking

Index	Residue	AA	Distance	Angle	Offset	Stacking Type	Ligand Atoms
1	296A	TYR	3.46	9.33	0.39	P	10, 13, 14, 17, 18, 21
2	296A	TYR	4.67	60.80	1.71	T	6, 8, 9, 11, 12, 16

Docking Results of Ligand 9

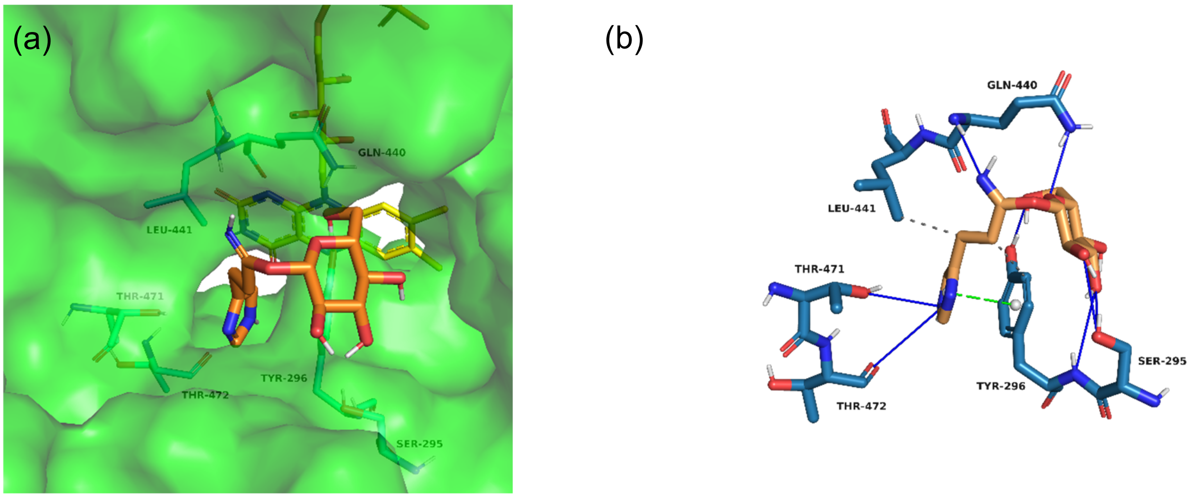

Figure S9. Interactions between **9** and SmTGR displayed as docking pose (a) and PLIP analysis (b). Ligand **9** occupies the doorstop pocket with its imidazole ring. Although its NH is not directly addressed by an amino acid, it could interact with the water molecule 760 located in this subpocket (toggle mode was used during docking, allowing water in the binding pocket to be taken into account). The main chain GLN440-NH is addressed through the nitrogen of the carboximidate moiety. The glucose unit of ligand **9** extends into the subpocket wherein phosphate and sugar residues are stabilized in the NADPH-bound state. In the case of ligand **9**, hydrogen bonds are formed between the ring-bound oxygen and GLN440-NH2 as well as between the glycosidic hydroxy groups and TYR296 and SER295. Orange sticks – insect molecule, yellow sticks – FAD, dashed grey lines – hydrophobic interactions, blue lines – hydrogen bonds, dashed green lines – π-stacking (parallel), orange dashed lines – salt bridge.

Table S9: Interactions between **9** and SmTGR as observed in the PLIP analysis.

Hydrophobic Interactions

Index	Residue	AA	Distance	Ligand Atom	Protein Atom
1	296A	TYR	3.78	14	4498
2	441A	LEU	3.74	16	6741

Hydrogen Bonds

Index	Residue	AA	Distance H-A	Distance D-A	Donor Angle	Protein donor?	Side chain	Donor Atom	Acceptor Atom
1	295A	SER	2.59	3.39	160.88	✓	✓	4483 [O3]	9 [O3]
2	295A	SER	2.48	3.39	155.63	×	✓	9 [O3]	4483 [O3]
3	295A	SER	2.01	2.78	134.01	×	✓	15 [O3]	4483 [O3]
4	296A	TYR	2.95	3.73	151.16	✓	×	4489 [Nam]	15 [O3]
5	296A	TYR	1.82	2.65	169.76	✓	✓	4500 [O3]	12 [O3]
6	296A	TYR	1.82	2.65	140.60	×	✓	12 [O3]	4500 [O3]
7	440A	GLN	2.45	3.31	171.40	✓	×	6717 [Nam]	13 [N3]
8	440A	GLN	2.36	3.20	164.10	✓	✓	6724 [Nam]	1 [O3]
9	471A	THR	3.50	3.80	105.16	✓	✓	7200 [O3]	21 [Npl]
10	472A	THR	3.44	4.07	122.40	×	×	21 [Npl]	7211 [O2]

π-Stacking

Index	Residue	AA	Distance	Angle	Offset	Stacking Type	Ligand Atoms
1	296A	TYR	3.43	13.95	0.63	P	17, 18, 19, 20, 21

Docking Results of Ligand 10

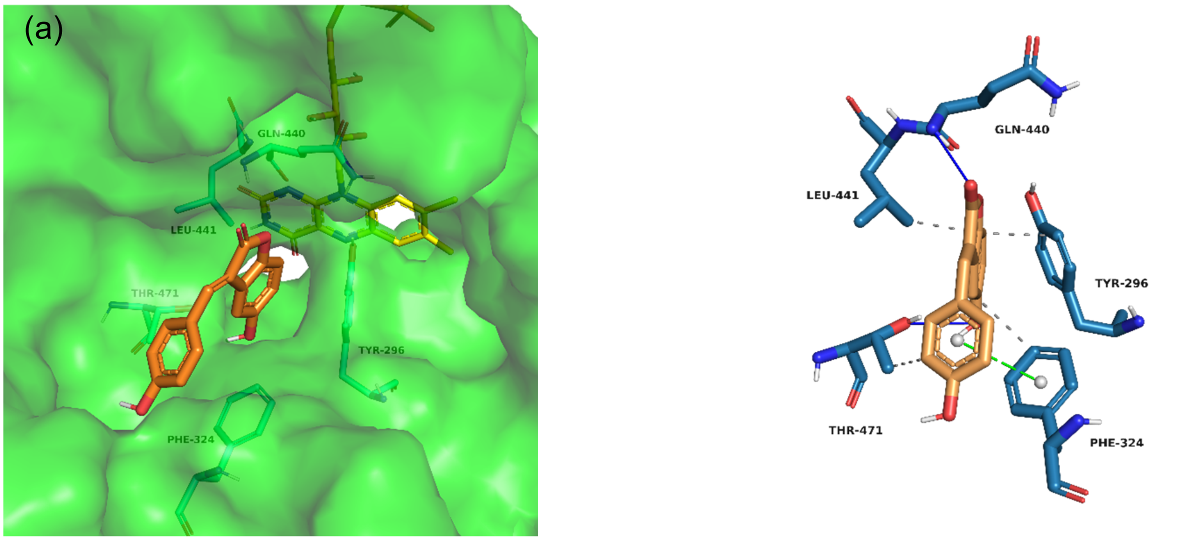

Figure S10. Interactions between **10** and SmTGR displayed as docking pose (a) and PLIP analysis (b). The part of the molecule occupying the doorstop pocket is predicted to interact with THR471 by hydrogen bonding and π-π interactions (PHE324) as well as hydrophobic interactions (TYR296, PHE324). Analysis of ligand **10** revealed a hydrogen bond between the carbonyl oxygen and the main chain GLN440-NH. Additionally, ligand **10** shows a π-π interaction (parallel displaced) with PHE324 and thus additionally occupies the HEPE subpocket. Orange sticks – insect molecule, yellow sticks – FAD, dashed grey lines – hydrophobic interactions, blue lines – hydrogen bonds, dashed green lines – π-stacking (parallel), orange dashed lines – salt bridge.

Table S10: Interactions between **10** and SmTGR as observed in the PLIP analysis.

Hydrophobic Interactions

Index	Residue	AA	Distance	Ligand Atom	Protein Atom
1	296A	TYR	3.27	8	4483
2	324A	PHE	3.40	9	4908
3	441A	LEU	3.03	8	6726
4	471A	THR	3.38	17	7184

Hydrogen Bonds

Index	Residue	AA	Distance H-A	Distance D-A	Donor Angle	Protein donor?	Side chain	Donor Atom	Acceptor Atom
1	440A	GLN	2.15	2.97	158.30	✓	×	6702 [Nam]	1 [O2]
2	471A	THR	2.46	2.88	112.22	✓	✓	7185 [O3]	15 [O2]
3	471A	THR	2.19	2.88	126.52	×	✓	15 [O2]	7185 [O3]

π-Stacking

Index	Residue	AA	Distance	Angle	Offset	Stacking Type	Ligand Atoms
1	324A	PHE	4.01	16.67	1.54	P	10, 13, 14, 16, 17, 18

Comparison of docking pose of 1,8-naphthyridine-2-carboxylate (A1) with the crystal structure

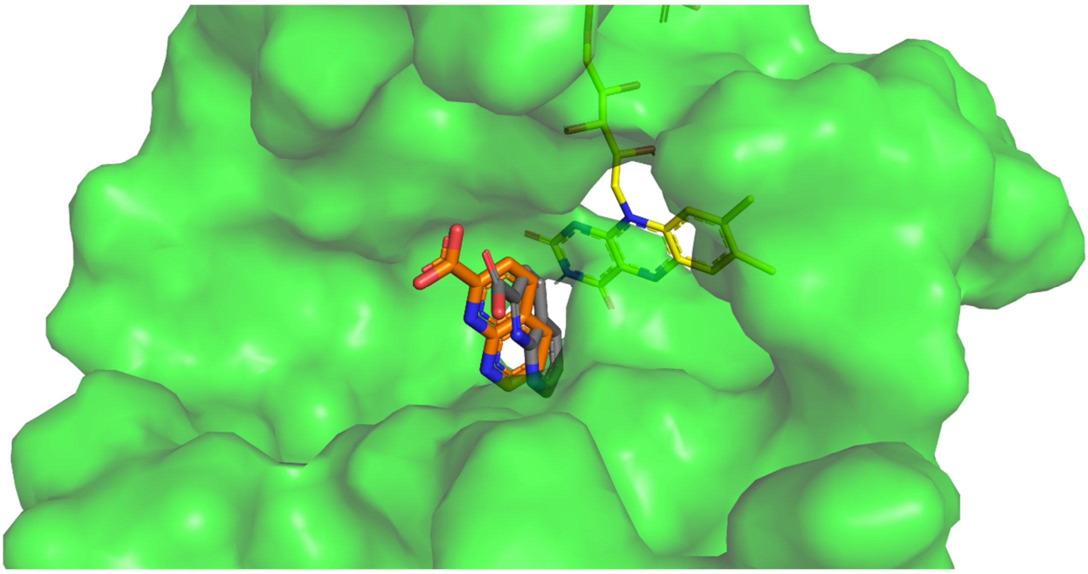

Figure S11. Docking of 1,8-naphthyridine-2-carboxylate (**A1**) in SmTGR to validate the docking procedure. The binding pose obtained in the docking (grey sticks) is comparable to **A1** (orange sticks) in the crystal structure (6FP4).
